# Effects of Lifestyle Interventions on Cardiovascular Disease Risk and Risk Factors Among Individuals at High Risk for Type 2 Diabetes: Protocol for a Systematic Review and Meta-Analysis of Randomized Controlled Trials

**DOI:** 10.2196/53517

**Published:** 2024-06-27

**Authors:** Getu Debalkie Demissie, Josephine Birungi, Tilahun Haregu, Sathish Thirunavukkarasu, Brian Oldenburg

**Affiliations:** 1 School of Psychology and Public Health La Trobe University Melbourne Australia; 2 Baker Heart and Diabetes Institute Melbourne Australia; 3 Melbourne School of Population and Global Health University of Melbourne Melbourne Australia; 4 Department of Family and Preventive Medicine School of Medicine Emory University Atlanta, GA United States; 5 Emory Global Diabetes Research Center Woodruff Health Sciences Center Emory University Atlanta, GA United States

**Keywords:** diabetes, prediabetes, cardiovascular disease, CVD, CVD risk, CVD risk factors, lifestyle interventions, systematic review, meta-analysis

## Abstract

**Background:**

Individuals at high risk for type 2 diabetes are also at an increased risk for developing cardiovascular disease (CVD). Although there are separate trials examining the effects of lifestyle interventions on absolute CVD risk among people at high risk for type 2 diabetes, a comprehensive evidence synthesis of these trials is lacking.

**Objective:**

We will systematically synthesize the evidence on the effects of lifestyle interventions in reducing absolute CVD risk and CVD risk factors among people at high risk for type 2 diabetes.

**Methods:**

We adhered to the PRISMA-P (Preferred Reporting Items for Systematic Review and Meta-Analysis Protocols) statement in reporting the details of this protocol. Randomized controlled trials of diabetes prevention that examined the effects of lifestyle interventions for at least 6 months on absolute CVD risk and CVD risk factors among individuals at high risk for type 2 diabetes will be eligible. We will systematically search the MEDLINE, Embase, PsycINFO, CENTRAL, and Scopus databases and ClinicalTrials.gov using a mix of Medical Subject Headings and text words. Two authors will independently screen the abstract and title of the articles retrieved from the search, followed by full-text reviews using the inclusion and exclusion criteria and data extraction from the eligible studies. Article screening and data extraction will be performed in the Covidence software. The primary outcome will be the changes in absolute 10-year CVD risk, as estimated by risk prediction models. The secondary outcomes are the changes in CVD risk factors, including behavioral, clinical, biochemical, and psychosocial risk factors, and incidence of type 2 diabetes.

**Results:**

An initial database search was conducted in July 2023. After screening 1935 articles identified through the database search, 42 articles were considered eligible for inclusion. It is anticipated that the study findings will be submitted for publication in a peer-reviewed journal by the end of 2024.

**Conclusions:**

This study will provide up-to-date, systematically synthesized evidence on the effects of lifestyle interventions on absolute CVD risk and CVD risk factors among individuals at high risk for type 2 diabetes.

**Trial Registration:**

PROSPERO CRD42023429869; https://tinyurl.com/59ajy7rw

**International Registered Report Identifier (IRRID):**

DERR1-10.2196/53517

## Introduction

Cardiovascular diseases (CVDs), mainly ischemic heart disease and stroke, are the leading causes of premature deaths and disability among adults worldwide [1]. In 2019, CVDs accounted for an estimated 18.6 million deaths and 34.4 million years lived with disability [1].

Hyperglycemia is a well-established risk factor for CVDs [2,3]. Individuals with a high risk of developing type 2 diabetes, such as those with prediabetes, also face an elevated risk of experiencing CVD events [4], with higher incidence rates than the general population [5,6]. Consequently, it is imperative to implement interventions for the primary prevention of CVDs among those at high risk for type 2 diabetes [7,8].

Behavioral risk factors such as an unhealthy diet, heavy alcohol drinking, and physical inactivity lead to increased BMI, plasma glucose, and serum lipids and the development of CVD events [1,9]. Studies have also shown that psychosocial factors, such as anxiety, depression, stress, social isolation, and the lack of social support, can independently influence absolute CVD risk [10,11].

Assessing an individual’s CVD risk is the initial step in the primary prevention of CVDs [12]. The international clinical guidelines from the World Health Organization (WHO) and the American Heart Association recommend estimating a person’s 10-year absolute risk of developing CVD events using risk scores that quantify the cumulative impact of multiple risk factors [12,13]. The predicted 10-year CVD risk not only informs treatment strategies but also assists in assessing the effectiveness of those strategies [14]. Furthermore, treatment options vary based on the levels of CVD risk. For example, the European Society of Cardiology guidelines recommend using the Systematic Coronary Risk Evaluation 2 (SCORE2) algorithm, which includes age, sex, current smoking, systolic blood pressure (BP), total cholesterol, and high-density lipoprotein cholesterol. This algorithm helps determine an individual’s CVD risk category as “low-to-moderate,” “high,” or “very high” [9,14]. Although the guidelines advocate for smoking cessation and the adoption of healthy lifestyle choices for individuals in any risk category, treatment for high systolic BP and cholesterol is specifically recommended for those with “high” or “very high” scores [15].

Randomized controlled trials (RCTs) focusing on adopting a healthy diet, increasing physical activity, quitting smoking, and addressing stress and other psychosocial risk factors have shown significant reductions in absolute CVD risk [16,17] and CVD events [8,12] among individuals at high risk for type 2 diabetes. However, there have also been lifestyle-based RCTs that did not report significant results [18]. These discrepancies in the outcomes of trials underscore the importance of conducting a systematic review to provide a comprehensive summary of the existing evidence. Previous systematic reviews and meta-analyses on reduction in absolute CVD risk with lifestyle interventions included people with type 2 diabetes, the general population, or high-risk groups for CVD (eg, individuals with hypertension or obesity) [17,19], but not those at high risk for type 2 diabetes. Notably, although previous systematic reviews have examined the effects of lifestyle-based diabetes prevention trials on traditional CVD risk factors (eg, obesity and high total cholesterol) [17,19-21], none specifically examined the impact of such trials on psychosocial risk factors. This is despite studies showing that psychosocial risk factors influence absolute CVD risk and risk factors [13,22].

The proposed systematic review will thus fill a substantial gap in the existing literature by focusing on individuals at high risk for type 2 diabetes and assessing the effects of lifestyle interventions on absolute CVD risk and not only traditional CVD risk factors but also psychosocial risk factors.

## Methods

### Overview

We adhered to the PRISMA-P (Preferred Reporting Items for Systematic Review and Meta-Analysis Protocols) statement in reporting the details of this protocol [23]. The PRISMA-P checklist has been included in Multimedia Appendix 1 [23]. The proposed systematic review and meta-analysis will be conducted per the *Cochrane Handbook for Systematic Reviews of Interventions* [24] and will follow the PRISMA (Preferred Reporting Items for Systematic Reviews and Meta-Analyses) guideline for reporting [25]. This review has been registered in PROSPERO (CRD42023429869).

### Inclusion Criteria

The following PICOS (Population, Intervention, Comparator, Outcome, and Study Design) framework [26] will be considered to determine the eligibility of studies (Table 1).

**Table 1:** PICOS (Population, Intervention, Comparator, Outcome, and Study Design) framework.

Criteria	Description
Population	Adults (aged ≥18 years) at high risk of developing type 2 diabetes: Impaired fasting glucose, defined by the ADA^a^ criteria (FPG^b^ 100 mg/dL [5.6 mmol/L] to 125 mg/dL [6.9 mmol/L]) [27] or the WHO^c^ criteria (FPG 110 mg/dL [6.1 mmol/L] to 125 mg/dL [6.9 mmol/L]) [28] or Impaired glucose tolerance, defined as 2-hour plasma glucose 140 mg/dL (7.8 mmol/L) to 199 mg/dL (11.0 mmol/L) on a 75-g oral glucose tolerance test [27] or Elevated hemoglobin A_1c_: 5.7%-6.4% (39-46 mmol/mol) [29] or 6.0%-6.4% (42-47 mmol/mol) [30] or High diabetes risk scores: for example, the Indian Diabetes Risk Score ≥60 [31], the Finnish Diabetes Risk Score ≥12 [32], the ADA risk test ≥5 points [33], and the Leicester Risk Assessment Score ≥16 [34]
Intervention	Structured lifestyle intervention programs for 6 months or more that aimed to improve diet quality, increase physical activity, help participants quit smoking, reduce alcohol consumption, or provide advice on other healthy lifestyle choices
Comparator	Usual or standard care with or without minimal intervention (eg, providing health education booklets)
Outcome	*Primary outcome*: Changes in absolute 10-year CVD^d^ risk, as estimated by risk prediction models, and the incidence of fatal and nonfatal CVD events (myocardial infarction, angina, stroke, peripheral artery disease, need for coronary bypass grafting, or heart failure) *Secondary outcomes*: Changes in behavioral, clinical, biochemical, and psychosocial risk factors and incidence of type 2 diabetes Behavioral risk factors include an unhealthy diet (includes poor diet quality or increased consumption of processed foods that are high in sugars and saturated fat), physical inactivity, alcohol consumption, tobacco use, and sleep time and quality Clinical measures are weight, BMI, waist circumference, and systolic and diastolic blood pressure Biochemical measures are FPG, hemoglobin A_1c_, total cholesterol, high-density lipoprotein cholesterol, low-density lipoprotein cholesterol, and triglycerides Psychosocial measures are stress, depression, and anxiety
Study design	Randomized controlled trials

^a^ADA: American Diabetes Association.

^b^FPG: fasting plasma glucose.

^c^WHO: World Health Organization.

^d^CVD: cardiovascular disease.

### Exclusion Criteria

Studies conducted among individuals with diagnosed type 2 diabetes or gestational diabetes will be excluded. Studies testing pharmacological or surgical interventions, non-RCT studies, and articles not published in English will also be excluded.

### Data Sources and Search Strategy

Bibliographic databases, such as Ovid MEDLINE, Embase, CENTRAL, PsycINFO, and Scopus, and ClinicalTrials.gov will be searched for articles reporting on the effects of lifestyle interventions on absolute CVD risk and CVD risk factors among individuals at high risk of developing type 2 diabetes. Our search strategy will include a combination of Medical Subject Headings and free-text terms. The search terms are “cardiovascular disease,” “lifestyle interventions,” “diet,” “physical activity,” “prediabetes,” “impaired fasting glucose,” “impaired glucose tolerance,” “diabetes risk score,” “diabetes prevention,” “cardiovascular disease risk score,” “cardiovascular risk factors,” “psychosocial risk factors,” “stress,” “depression,” and “anxiety.” A comprehensive search strategy for each bibliographic database will be developed in consultation with a librarian and experts in the field of diabetes and CVD. As an example, the search strategy for MEDLINE is given in Multimedia Appendix 2.

### Study Selection

All identified studies will be exported to the Covidence software (Veritas Health Innovation), and duplicates will be removed. Two independent reviewers will screen the titles and abstracts of eligible studies. The full-text reviews will be performed against the inclusion and exclusion criteria by the same 2 reviewers. Disagreements arising between the reviewers at any stage of study selection will be resolved by discussion or consultation with a third reviewer.

### Data Extraction

Two independent reviewers will extract data from eligible studies using a template designed by the Covidence software. The data extracted will pertain to specific details about study participants, lifestyle interventions, study methods (eg, study setting and follow-up time), and outcomes. Any reviewer conflicts will be resolved through discussion or consultation with a third reviewer. The authors of the papers will be contacted to obtain missing or additional data if needed.

### Risk of Bias and the Certainty of Evidence

Two reviewers will independently assess the potential sources of bias specific to RCTs using the revised Cochrane risk-of-bias tool for randomized controlled trials (RoB 2) [35]. The GRADE (Grading of Recommendations, Assessment, Development and Evaluation) framework [36] will be used to determine the certainty of the evidence. Consensus between the reviewers will be achieved by discussion or consultation with a third reviewer.

### Data Synthesis

After the selection of eligible studies and data extraction, the included studies will be described in a narrative form focusing on the following: income status of the countries (high-income or low- and middle-income countries) where the studies were conducted, study setting (community, hospital, or workplace), age and sex distribution of the study participants, components of the lifestyle intervention programs (eg, diet, physical activity, sleep, stress, and smoking), duration of the interventions, duration of follow-up, and outcomes.

Where possible, outcomes data across studies will be pooled using the DerSimonian-Laird random-effects models for meta-analysis [37]. Effect sizes will be expressed as risk ratios (for categorical variables) and standardized mean differences (for continuous variables) with 95% CIs. The degree of between-study heterogeneity will be assessed using the Cochran *Q* test (*P*<.01 for heterogeneity) and Higgins *I*^2^ statistic (low: <25%, moderate: 25%-50%, and high: >50%) [38]. *I*^2^ quantifies the percentage of variability in effect estimates due to heterogeneity rather than sampling error [24]. Subgroup analyses will be considered if there are sufficient data to examine the effects of lifestyle interventions by types of high-risk individuals (impaired fasting glucose, impaired glucose tolerance, elevated hemoglobin A_1c_, and high diabetes risk score) and different prediabetes definitions. Publication bias will be assessed by funnel plots [39] and Egger test [40] if 10 or more studies are included in the meta-analysis [38]. A 2-sided *P*<.05 will be considered statistically significant. Analyses will be performed using RevMan (version 5.4.1; The Cochrane Collaboration) and Stata software (version 17.0; StataCorp).

### Ethical Considerations

This is a systematic review and meta-analysis based on previously published studies and will not involve individuals. Therefore, ethical approval is not required.

## Results

**Figure 1:**
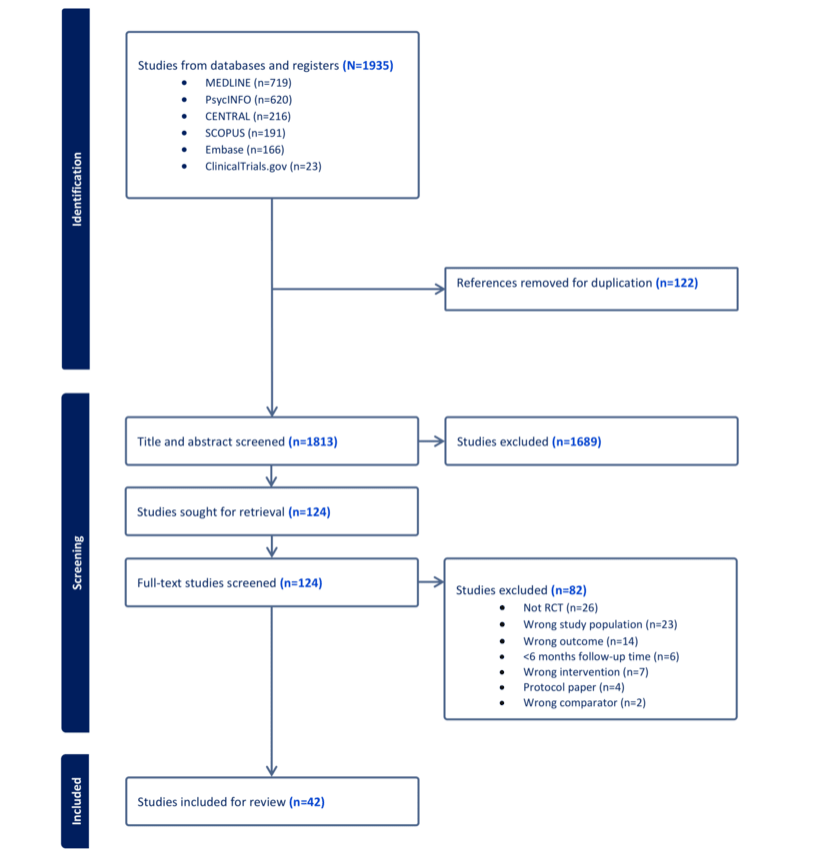
PRISMA (Preferred Reporting Items for Systematic Reviews and Meta-Analyses) flowchart of the study selection. RCT: randomized controlled trial.

Figure 1 shows the PRISMA flowchart. An initial database search was conducted in July 2023, identifying a total of 1935 articles. After removing 122 duplicates, 1813 articles remained. The titles and abstracts of these articles were screened, resulting in 124 studies selected for full-text review. Following this review, 82 studies were excluded for various reasons. Finally, 42 studies were included for data extraction. Currently, 2 independent reviewers are extracting data from these studies and conducting a quality assessment of the studies. We anticipate that the results of the study will be submitted for publication in a peer-reviewed journal by the end of 2024. The study findings will also be disseminated through presentations at scientific conferences and meetings.

## Discussion

### Novelty and Expected Findings

This study aims to provide up-to-date and summarized evidence on the effects of lifestyle interventions in reducing absolute CVD risk and CVD risk factors among individuals at high risk for type 2 diabetes. By specifically focusing on individuals at high risk for developing type 2 diabetes, this study aims to bridge a significant gap in the existing literature. Previous systematic reviews and meta-analyses have predominantly centered around people with established type 2 diabetes or the general population, making this study distinctive in its targeted approach.

### Limitations

One of the limitations of this study is that our search is specific to the English language; studies published in other languages may be missed, and this may lead to an incomplete representation of the available evidence. Although we used a comprehensive search strategy, if relevant studies are still missed during the search process, the review’s conclusions may be biased.

### Potential Implications

The study results are expected to provide valuable insights that can contribute to shaping future guidelines about the reduction of CVD risk and the prevention of CVD events.

## Appendix

Multimedia Appendix 1 PRISMA-P (Preferred Reporting Items for Systematic Review and Meta-Analysis Protocols) checklist.

Multimedia Appendix 2 Search strategy for MEDLINE.

## Abbreviations

BP: blood pressure
CVD: cardiovascular disease
GRADE: Grading of Recommendations, Assessment, Development and Evaluation
PICOS: Population, Intervention, Outcome, Comparator, and Study Design
PRISMA: Preferred Reporting Items for Systematic Reviews and Meta-Analyses
PRISMA-P: Preferred Reporting Items for Systematic Review and Meta-Analysis Protocols
SCORE2: Systematic Coronary Risk Evaluation 2
RCT: randomized controlled trial
WHO: World Health Organization

## Data Availability

All data generated and analyzed for this study are included in the paper and its supplementary files.

## References

[1] Roth GA, Mensah GA, Johnson CO, Addolorato G, Ammirati E, Baddour LM, Barengo NC, Beaton AZ, Benjamin EJ, Benziger CP, Bonny A, Brauer M, Brodmann M, Cahill TJ, Carapetis J, Catapano AL, Chugh SS, Cooper LT, Coresh J, Criqui M, DeCleene N, Eagle KA, Emmons-Bell S, Feigin VL, Fernández-Solà J, Fowkes G, Gakidou E, Grundy SM, He FJ, Howard G, Hu F, Inker L, Karthikeyan G, Kassebaum N, Koroshetz W, Lavie C, Lloyd-Jones D, Lu HS, Mirijello A, Temesgen AM, Mokdad A, Moran AE, Muntner P, Narula J, Neal B, Ntsekhe M, Moraes de Oliveira G, Otto C, Owolabi M, Pratt M, Rajagopalan S, Reitsma M, Ribeiro ALP, Rigotti N, Rodgers A, Sable C, Shakil S, Sliwa-Hahnle K, Stark B, Sundström J, Timpel P, Tleyjeh IM, Valgimigli M, Vos T, Whelton PK, Yacoub M, Zuhlke L, Murray C, Fuster V (2020). Global burden of cardiovascular diseases and risk factors, 1990-2019: update from the GBD 2019 study. J Am Coll Cardiol.

[2] Levitan EB, Song Y, Ford ES, Liu S (2004). Is nondiabetic hyperglycemia a risk factor for cardiovascular disease? a meta-analysis of prospective studies. Arch Intern Med.

[3] Danaei G, Lawes CMM, Vander Hoorn S, Murray CJL, Ezzati M (2006). Global and regional mortality from ischaemic heart disease and stroke attributable to higher-than-optimum blood glucose concentration: comparative risk assessment. Lancet.

[4] Huang Y, Cai X, Mai W, Li M, Hu Y (2016). Association between prediabetes and risk of cardiovascular disease and all cause mortality: systematic review and meta-analysis. BMJ.

[5] Cheng YJ, Gregg EW, Geiss LS, Imperatore G, Williams DE, Zhang X, Albright AL, Cowie CC, Klein R, Saaddine JB (2009). Association of A1c and fasting plasma glucose levels with diabetic retinopathy prevalence in the U.S. population: implications for diabetes diagnostic thresholds. Diabetes Care.

[6] Ford ES, Zhao G, Li C (2010). Pre-diabetes and the risk for cardiovascular disease: a systematic review of the evidence. J Am Coll Cardiol.

[7] Li G, Zhang P, Wang J, An Y, Gong Q, Gregg EW, Yang W, Zhang B, Shuai Y, Hong J, Engelgau MM, Li H, Roglic G, Hu Y, Bennett PH (2014). Cardiovascular mortality, all-cause mortality, and diabetes incidence after lifestyle intervention for people with impaired glucose tolerance in the Da Qing Diabetes Prevention Study: a 23-year follow-up study. Lancet Diabetes Endocrinol.

[8] Perreault L, Temprosa M, Mather KJ, Horton E, Kitabchi A, Larkin M, Montez MG, Thayer D, Orchard TJ, Hamman RF, Goldberg RB (2014). Regression from prediabetes to normal glucose regulation is associated with reduction in cardiovascular risk: results from the Diabetes Prevention Program outcomes study. Diabetes Care.

[9] Visseren FLJ, Mach F, Smulders YM, Carballo D, Koskinas KC, Bäck M, Benetos A, Biffi A, Boavida J, Capodanno D, Cosyns B, Crawford C, Davos CH, Desormais I, Di Angelantonio E, Franco OH, Halvorsen S, Hobbs FDR, Hollander M, Jankowska EA, Michal M, Sacco S, Sattar N, Tokgozoglu L, Tonstad S, Tsioufis KP, van Dis I, van Gelder IC, Wanner C, Williams B (2022). 2021 ESC Guidelines on cardiovascular disease prevention in clinical practice. Eur J Prev Cardiol.

[10] (2009). 2008-2013 Action plan for the global strategy for the prevention and control of noncommunicable diseases: prevent and control cardiovascular diseases, cancers, chronic respiratory diseases and diabetes. World Health Organization.

[11] Rugulies R (2002). Depression as a predictor for coronary heart disease. a review and meta-analysis. Am J Prev Med.

[12] Arnett DK, Blumenthal RS, Albert MA, Buroker AB, Goldberger ZD, Hahn EJ, Himmelfarb CD, Khera A, Lloyd-Jones D, McEvoy JW, Michos ED, Miedema MD, Muñoz D, Smith SC, Virani SS, Williams KA, Yeboah J, Ziaeian B (2019). 2019 ACC/AHA guideline on the primary prevention of cardiovascular disease: a report of the American College of Cardiology/American Heart Association Task Force on Clinical Practice Guidelines. Circulation.

[13] (2007). Prevention of cardiovascular disease: guidelines for assessment and management of total cardiovascular risk. World Health Organization.

[14] SCORE2 Working Group, ESC Cardiovascular Risk Collaboration (2021). SCORE2 risk prediction algorithms: new models to estimate 10-year risk of cardiovascular disease in Europe. Eur Heart J.

[15] Weintraub WS, Daniels SR, Burke LE, Franklin BA, Goff DC, Hayman LL, Lloyd-Jones D, Pandey DK, Sanchez EJ, Schram AP, Whitsel LP (2011). Value of primordial and primary prevention for cardiovascular disease: a policy statement from the American Heart Association. Circulation.

[16] Lotfaliany M, Sathish T, Shaw J, Thomas E, Tapp RJ, Kapoor N, Thankappan KR, Oldenburg B (2020). Effects of a lifestyle intervention on cardiovascular risk among high-risk individuals for diabetes in a low- and middle-income setting: secondary analysis of the Kerala Diabetes Prevention Program. Prev Med.

[17] Limbachia J, Ajmeri M, Keating BJ, de Souza RJ, Anand SS (2022). Effects of lifestyle interventions on cardiovascular risk factors in South Asians: a systematic review and meta-analysis. BMJ Open.

[18] Davies MJ, Gray LJ, Ahrabian D, Carey M, Farooqi A, Gray A, Goldby S (2017). A community-based primary prevention programme for type 2 diabetes mellitus integrating identification and lifestyle intervention for prevention: a cluster randomised controlled trial. Programme Grants for Applied Research, No. 5.2.

[19] Zhang X, Devlin HM, Smith B, Imperatore G, Thomas W, Lobelo F, Ali MK, Norris K, Gruss S, Bardenheier B, Cho P, Garcia de Quevedo I, Mudaliar U, Jones CD, Durthaler JM, Saaddine J, Geiss LS, Gregg EW (2017). Effect of lifestyle interventions on cardiovascular risk factors among adults without impaired glucose tolerance or diabetes: a systematic review and meta-analysis. PLoS One.

[20] Shirinzadeh M, Afshin-Pour B, Angeles R, Gaber J, Agarwal G (2019). The effect of community-based programs on diabetes prevention in low- and middle-income countries: a systematic review and meta-analysis. Global Health.

[21] Hopper I, Billah B, Skiba M, Krum H (2011). Prevention of diabetes and reduction in major cardiovascular events in studies of subjects with prediabetes: meta-analysis of randomised controlled clinical trials. Eur J Cardiovasc Prev Rehabil.

[22] Rosengren A, Hawken S, Ounpuu S, Sliwa K, Zubaid M, Almahmeed WA, Blackett KN, Sitthi-amorn C, Sato H, Yusuf S (2004). Association of psychosocial risk factors with risk of acute myocardial infarction in 11119 cases and 13648 controls from 52 countries (the INTERHEART study): case-control study. Lancet.

[23] Shamseer L, Moher D, Clarke M, Ghersi D, Liberati A, Petticrew M, Shekelle P, Stewart LA (2015). Preferred Reporting Items for Systematic Review and Meta-Analysis Protocols (PRISMA-P) 2015: elaboration and explanation. BMJ.

[24] Higgins JPT, Thomas J, Chandler J, Cumpston M, Li T, Page MJ, Welch VA (2019). Cochrane Handbook for Systematic Reviews of Interventions.

[25] Page MJ, McKenzie JE, Bossuyt PM, Boutron I, Hoffmann TC, Mulrow CD, Shamseer L, Tetzlaff JM, Akl EA, Brennan SE, Chou R, Glanville J, Grimshaw JM, Hróbjartsson A, Lalu MM, Li T, Loder EW, Mayo-Wilson E, McDonald S, McGuinness LA, Stewart LA, Thomas J, Tricco AC, Welch VA, Whiting P, Moher D (2021). The PRISMA 2020 statement: an updated guideline for reporting systematic reviews. BMJ.

[26] Amir-Behghadami M, Janati A (2020). Population, Intervention, Comparison, Outcomes and Study (PICOS) design as a framework to formulate eligibility criteria in systematic reviews. Emerg Med J.

[27] ElSayed NA, Aleppo G, Aroda VR, Bannuru RR, Brown FM, Bruemmer D, Collins BS, Hilliard ME, Isaacs D, Johnson EL, Kahan S, Khunti K, Leon J, Lyons SK, Perry ML, Prahalad P, Pratley RE, Seley JJ, Stanton RC, Gabbay RA (2023). 2. Classification and diagnosis of diabetes: Standards of Care in Diabetes-2023. Diabetes Care.

[28] (2006). Definition and diagnosis of diabetes mellitus and intermediate hyperglycaemia. World Health Organization.

[29] American Diabetes Association (2021). 2. Classification and diagnosis of diabetes: Standards of Care in Diabetes-2021. Diabetes Care.

[30] Gillett MJ (2009). International Expert Committee report on the role of the A1c assay in the diagnosis of diabetes: Diabetes Care 2009; 32(7): 1327-1334. Clin Biochem Rev.

[31] Mohan V, Deepa R, Deepa M, Somannavar S, Datta M (2005). A simplified Indian Diabetes Risk Score for screening for undiagnosed diabetic subjects. J Assoc Physicians India.

[32] Lindström J, Tuomilehto J (2003). The Diabetes Risk Score: a practical tool to predict type 2 diabetes risk. Diabetes Care.

[33] American Diabetes Association Professional Practice Committee (2024). 2. Diagnosis and classification of diabetes: Standards of Care in Diabetes-2024. Diabetes Care.

[34] Gray LJ, Taub NA, Khunti K, Gardiner E, Hiles S, Webb DR, Srinivasan BT, Davies MJ (2010). The Leicester Risk Assessment score for detecting undiagnosed type 2 diabetes and impaired glucose regulation for use in a multiethnic UK setting. Diabet Med.

[35] Sterne JAC, Savović J, Page MJ, Elbers RG, Blencowe NS, Boutron I, Cates CJ, Cheng HY, Corbett MS, Eldridge SM, Emberson JR, Hernán MA, Hopewell S, Hróbjartsson A, Junqueira DR, Jüni P, Kirkham JJ, Lasserson T, Li T, McAleenan A, Reeves BC, Shepperd S, Shrier I, Stewart LA, Tilling K, White IR, Whiting PF, Higgins JPT (2019). RoB 2: a revised tool for assessing risk of bias in randomised trials. BMJ.

[36] Guyatt GH, Oxman AD, Vist GE, Kunz R, Falck-Ytter Y, Alonso-Coello P, Schünemann HJ (2008). GRADE: an emerging consensus on rating quality of evidence and strength of recommendations. BMJ.

[37] DerSimonian R, Laird N (1986). Meta-analysis in clinical trials. Control Clin Trials.

[38] Deeks JJ, Higgins JPT, Altman DG (2023). Chapter 10: analysing data and undertaking meta-analyses. Cochrane.

[39] Lin L, Chu H (2018). Quantifying publication bias in meta-analysis. Biometrics.

[40] Egger M, Davey SG, Schneider M, Minder C (1997). Bias in meta-analysis detected by a simple, graphical test. BMJ.

