# Supplementary material for: Effects of Lifestyle Interventions on Cardiovascular Disease Risk and Risk Factors Among Individuals at High Risk for Type 2 Diabetes: Protocol for a Systematic Review and Meta-Analysis of Randomized Controlled Trials
Source: JMIR Res Protoc. 2024 Jun 27;13:e53517. doi: 10.2196/53517 (PMC11240064; doi:10.2196/53517)
Supplement: Multimedia Appendix 2 [file resprot_v13i1e53517_app2.docx]

**Multimedia Appendix 2**

**Search strategy for MEDLINE**

| 1. Lifestyle intervention.mp. 2. lifestyle modification.mp. 3. lifestyle change.mp. 4. Dietary modification.mp. 5. Structured lifestyle intervention.mp. 6. healthy diet advice/or councelling.ti,ab. 7. physical activity.mp. 8. Exercise.mp. 9. structured lifestyle intervention .ti,ab. 10. non-pharmacologic* intervention.ti,ab. 11. diabet* prevention.ti,ab,kf. 12. 1 or 2 or 3 or 4 or 5 or 6 or 7 or 8 or 9 or 10 or 11 |
| --- |
| 1. Prediabtes.mp. 2. High-risk individuals.ti,ab. 3. Impaired fasting glucose.mp. 4. Impaired glucose tolerance.mp. 5. Elevated haemoglobin A1C 6. 13 or 14 or 15 or 16 or 17 |
| 1. Cardiovascular risk reduction.mp. 2. Cardiovascular risk score.mp. 3. Cardiovascular risk score.ti,ab. 4. Framingham Risk Score.mp. 5. Framingham Risk Score.ti,ab. 6. Cardiovascular diseases/ or myocardial infarction/ or heart diseases/stroke.ti,ab. 7. Cardiovascular events /or cardiovascular morbidity/or cardiovascular mortality.ti,ab. 8. 19 or 20 or 21 or 22 or 23 or 24 or 25 |
| 1. Cardiovascular risk factors.ti,ab. 2. Overweight/ or Body Mass Index/ or Obesity/.mp. 3. Cholesterol/or HDL/or/LDL/or Triglyceride/or.ti,ab. 4. Systolic blood pressure/or Diastolic blood pressure or.ti,ab. 5. 27 or 28 or 29 or 30 |
| 1. Psychosocial risk factors.ti,ab. 2. Stress. ti,ab. 3. Depression. ti,ab. 4. Anxiety .ti,ab. 5. 32 or 33 or 34 or 35 |
| 1. 12 AND 18 AND 26 AND 31 AND 36 |
